# *P. vivax* Malaria and Dengue Fever Co-infection: A Cross-Sectional Study in the Brazilian Amazon

**DOI:** 10.1371/journal.pntd.0003239

**Published:** 2014-10-23

**Authors:** Belisa M. L. Magalhães, André M. Siqueira, Márcia A. A. Alexandre, Marcela S. Souza, João B. Gimaque, Michele S. Bastos, Regina M. P. Figueiredo, Gisely C. Melo, Marcus V. G. Lacerda, Maria P. G. Mourão

**Affiliations:** 1 Universidade do Estado do Amazonas, Manaus, Brazil; 2 Fundação de Medicina Tropical Dr. Heitor Vieira Dourado, Manaus, Brazil; The George Washington University Medical Center, United States of America

## Abstract

**Background:**

Malaria and dengue are the most prevalent vector-borne diseases worldwide and represent major public health problems. Both are endemic in tropical regions, propitiating co-infection. Only few co-infection cases have been reported around the world, with insufficient data so far to enhance the understanding of the effects of co-infection in the clinical presentation and severity.

**Methodology/Principal Findings:**

A cross-sectional study was conducted (2009 to 2011) in hospitalized patients with acute febrile syndrome in the Brazilian Amazon. All patients were submitted to thick blood smear and PCR for *Plasmodium* sp. detection, ELISA, PCR and NS1 tests for dengue, viral hepatitis, HIV and leptospirosis. In total, 1,578 patients were recruited. Among them, 176 (11.1%) presented *P. vivax* malaria mono-infection, 584 (37%) dengue fever mono-infection, and 44 (2.8%) were co-infected. Co-infected patients had a higher chance of presenting severe disease (vs. dengue mono-infected), deep bleeding (vs. *P. vivax* mono-infected), hepatomegaly, and jaundice (vs. dengue mono-infected).

**Conclusions/Significance:**

In endemic areas for dengue and malaria, jaundice (in dengue patients) and spontaneous bleeding (in malaria patients) should raise the suspicion of co-infection. Besides, whenever co-infection is confirmed, we recommend careful monitoring for bleeding and hepatic complications, which may result in a higher chance of severity, despite of the fact that no increased fatality rate was seen in this group.

## Author Summary

Malaria and dengue fever are typical diseases in tropical regions of developing countries; such as the Brazilian Amazon. They become serious problems in public health as they mostly affect vulnerable populations. Both diseases are mosquito-borne. These diseases present similar signs and symptoms. Brazil registers most of the malaria cases in the Amazon. The four dengue serotypes also circulate in this region. Similar to malaria, there are records of dengue outbreaks during the first months of the year, and isolated cases in the remaining months. Official records of malaria and dengue co-infection are infrequent in Brazil; however, we believe that this event is more frequent than usually reported. Our study detected high prevalence of the co-infection in the hospitalized patients infected with malaria or dengue in a tertiary health care unit, reference in the treatment of tropical and infectious diseases in Manaus, Amazonas, Brazil. We highlight the high likelihood of co-infected patients to present clinical complications. Besides, we observed that the presence of jaundice in dengue patients, and bleeding in malaria patients, are possible indications of co-infection. Therefore, this paper is useful to physicians working in the tropics, enabling the clinical suspicion of a not so rare condition.

## Introduction

Malaria and dengue fever are the most prevalent vector-borne diseases worldwide and represent major public health problems. Dengue epidemics have been reported in several countries; 500,000 people with severe dengue require hospitalization each year, and 2.5% of those affected die. Similarly, malaria is a life-threatening disease which was responsible for 627,000 deaths in 2012 [1], [2]. However, the occurrence of dengue and malaria co-infected patients is not well reported.

The dengue virus (DENV) is the major arbovirus responsible for human disease in Brazil. The four serotypes cause a variety of clinical presentation in humans, ranging from acute self-limited febrile illness to severe and fatal forms [3], [4]. Regarding malaria, the Brazilian Amazon reports 50% of episodes in the Americas [5]. In 2012, 241,806 cases were reported, with 86.9% of them due to *P. vivax* [6].

Malaria and dengue are endemic in similar tropical regions, and therefore, may result in the possibility of co-infection. Urban demographic expansion, deforestation and agricultural settlements in peri-urban areas, are known causes of the increase in the probability of concurrent infection of these two diseases [7].

Considering the endemicity of dengue and malaria in the Amazon [8], it is reasonable to envisage that the occurrence of concurrent infections would not be rare [9], [10]. However, due to non-systematic investigation of both diseases, only a few cases of malaria and dengue co-infection have been reported [11], [12]. In Brazil, for instance, a study performed in 2009 with 132 patients with vivax malaria found 11 co-infection episodes, all confirmed by molecular tests. These patients demonstrated severe manifestations, in particular hepatic injury [10]. The objective of the present study was to understand the interplay of both infections in a higher sample, and the impact on the clinical severity.

## Methods

### Ethical Statement

The study was approved by the Ethics Review Board of *Fundação de Medicina Tropical Dr. Heitor Vieira Dourado* (FMT-HVD, 2009/15243), Manaus, Brazil. All participants signed an informed consent.

### Study Design and Site

The study design was a cross-sectional study of patients hospitalized with acute febrile syndrome (history of fever in the past 10 days) from 2009 to 2011. The study was carried out in FMT-HVD, Manaus, capital of Amazonas State, Northern Brazil, where all four dengue serotypes co-circulate since 2008 and 95% of malaria cases result from *P. vivax* infection. FMT-HVD is a tertiary health care facility and a teaching and research center, which is the reference for infectious diseases in the region. Around 30% of all malaria cases reported in Manaus are assisted in this institution. During the study period, 14,884 cases of malaria and 6,302 cases of dengue fever were diagnosed in the hospital, from which 505 and 1,127, respectively, were hospitalized. In 2011, a dengue outbreak resulted in 5,400 cases reported at the FMT-HVD (∼10% of all reported cases in the state, based on data of surveillance system).

### Patients and Data Collection

During the study period, all hospitalized patients with acute febrile syndrome were considered eligible. If they signed the informed consent, they were included and submitted to malaria and dengue investigation. They were also searched for hepatitis A, B and C, HIV and leptospirosis. Abdominal ultrasound and chest X-rays were also performed when indicated. Other tests were requested at physicians' discretion.

Patients with *P. vivax* infection with primaquine-induced hemolysis (hemoglobin <10 g/dL and reticulocytes >1.5% or increased indirect bilirubin after starting primaquine) were also excluded from the analysis.

The diagnosis of vivax malaria was confirmed by real-time PCR. The diagnosis of dengue was made either by a positive serology (IgM) or a positive NS1 protein or a positive molecular test (RT-PCR), considering that every patient was tested by all the three methods. The Group C was defined as patients co-infected with both dengue and *P. vivax*. They were compared to two different groups: malaria mono-infection (Group A), and dengue mono-infection (Group B). Severity was classified and managed according to the World Health Organization (WHO) guidelines for dengue and malaria [2], [13].

### Laboratory Testing

Automatized blood biochemistry and whole blood count were performed systematically in all patients. The continuous variables used for analysis were the most altered throughout hospitalization. Walker's technique was used for thick blood smear [14]. The number of asexual parasites was counted in high magnification fields per 500 leukocytes and expressed as parasites per mm^3^. Real time polymerase chain reaction (qPCR) was performed to confirm *P. vivax* mono-infection. In brief, the extraction of total DNA from whole blood was performed using the QIAamp DNA Blood Mini Kit (Qiagen, USA), according to the manufacturer's protocol. The DNA was amplified in an Applied Biosystems 7500 Fast System (Applied Biosystems, USA) using primers and TaqMan fluorescence labeled probes for RT-PCR [15].

The DENV diagnosis was based on three methods: a) IgM antibodies (MAC-ELISA) detection [16]; b) detection of NS1 protein by Platelia Dengue NS1 Ag kit (Bio-Rad, France), and c) molecular diagnostics with the identification of viral serotype from the RT-PCR [17]. For extraction of viral RNA, mini kit QIAamp viral RNA (Qiagen, USA) was used, following the manufacturer's instructions. For the production of complementary DNA copy (cDNA) from RNA, AccessQuick kit RT-PCR System (Promega, USA) was used, according to the manufacturer's recommendations. The genomic region of dengue virus (DENV) was amplified by semi-nested PCR included genes C/prM.

Serological tests for leptospirosis (IgM) [18], HIV-1/HIV-2 [19], hepatitis A (anti-HAV IgM), hepatitis B (HBsAg), hepatitis C (anti-HCV), and hepatitis D (total anti-HDV), were based on commercial kits from Diasorin (Italy) and Bioeasy (Korea), following the manufacturers' instructions.

### Statistical Analysis

Demographics, clinical and laboratorial characteristics from the group of patients co-infected with dengue and malaria vivax were compared to the group of patients mono-infected with dengue and the group of patients mono-infected with malaria vivax. The association between categorical variables and the risk of co-infection (as the outcome variable) was performed by means of univariable logistic regression with the presentation of the odds ratios and 95% confidence intervals. The 95% confidence intervals (95% CI) are presented. Means and standard deviation (SD) of continuous variables with normal distributions were compared using the Student's T test; those variables with non-normal distribution (as assessed by the Kolmogorov-Smirnov test) were described using median and interquartile range (IQR) and compared using the Kruskal-Wallis test. All the analyses were performed using Stata v.11 (College Station, Texas, USA) [20].

## Results

**Figure 1:**
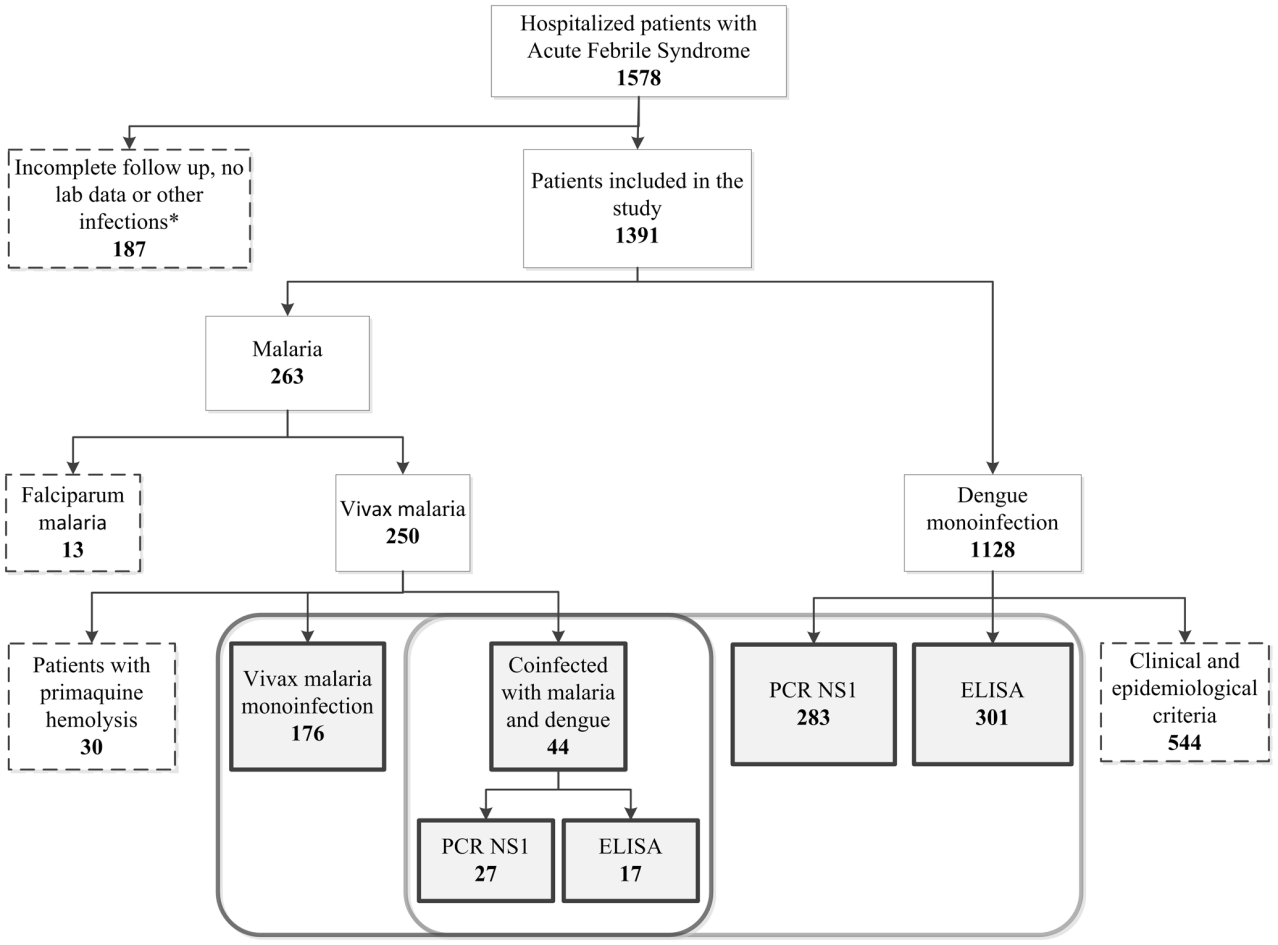
Flowchart of study participants. Gray boxes represent patients included in the analyses; Dashed boxes represent patients excluded.

From 2009 to 2011, 1,578 patients with acute febrile syndrome were hospitalized at the FMT-HVD. Among them, 176 (11.1%, 95% CI 9.6–12.7%) had vivax malaria mono-infection (Group A), 584 (37%, 95% CI 34.6–39.4%) had dengue fever mono-infection (Group B) and 44 (2.8%, 95% CI 2.0–3.6%) were co-infected with malaria and dengue (Group C). The prevalence of co-infected patients was 20% among patients with malaria and 7% among those with dengue (Figure 1).

As shown in Table 1, the characteristics across the groups are homogenous. It is important to highlight though that fewer children and pregnant women were included in the co-infected group.

**Table 1:** Demographic aspects and clinical characteristics of hospitalized patients with *P. vivax* malaria, dengue fever and *P. vivax* malaria and dengue fever co-infection admitted to a tertiary health center (Manaus, Brazil).

Variables	*P. vivax* (A)	Dengue fever (B)	Co-infection (C)	A×C		B×C	
				*OR (CI 95%)*	*p* b	*OR (CI 95%)*	*p* b
	N = 176 (%)	N = 584 (%)	N = 44 (%)				
**Male**	92 (52.3)	277 (47.4)	22 (50)	0.91 (0.47–1.76)	0.787	1.1 (0.6–2.0)	0.742
**Age (years)**							
**0–14**	29 (16.4)	60 (10.4)	3 (6.9)	1	0.078^c^	1	0.086^c^
**15–40**	105 (59.6)	355 (61.5)	22 (51.1)	2.02 (0.56–7.24)		1.23 (0.35–4.26)	
**41–60**	26 (14.7)	126 (21.8)	12 (27.9)	4.46 (1.13–17.58)		1.9 (0.51–7.0)	
**>60**	16 (9.1)	36 (6.2)	6 (13.9)	3.62 (0.79–16.48)		3.33 (0.78–14.15)	
**Pregnancy** a	14 (18.1)	8 (3.8)	4 (19.0)	1.05 (0.30–3.63)	0.928	11.4 (3.12–41.65)	<0.001
**Chronic diseases**	46 (26.4)	98 (24.8)	12 (27.2)	1.04 (0.49–2.19)	0.911	1.14 (0.56–2.29)	0.714
**Previous malaria**	67 (38.1)	-	15 (38.0)	0.84 (0.42–1.68)	0.626	-	-

a Total of 77 women in group A, 210 women in group B and 22 women in group C;

b p value of the Wald test derived from Logistic regression;

c p value from Student's T test.

Tables 2 and 3 compare clinical and laboratorial data between Group C and Groups A and B. Patients with the co-infection had a higher chance of presenting severe disease (OR 4.71, 95% CI: 2.37–9.34) according to WHO's criteria than those mono-infected with dengue (Table 2). Conversely, those with malaria mono-infection had less frequently severe disease than co-infected patients, but this was not statistically significant.

**Table 2:** Clinical description of hospitalized patients with *P. vivax* malaria, dengue fever and *P. vivax* malaria and dengue fever co-infection admitted to a tertiary health center (Manaus, Brazil).

Variables	*P.vivax* (A)	Dengue fever (B)	Co-infection (C)	A×C		B×C	
				*OR (CI 95%)*	*p* a	*OR (CI 95%)*	*p* a
	N = 176 (%)	N = 584 (%)	N = 44 (%)				
**Bleeding** *	54 (30.7)	306 (52.4)	24 (54.6)	2.7 (1.4–5.3)	0.004	1.1 (0.6–2)	0.783
**Superficial**	51 (29)	293 (50.2)	14 (31.8)	1.2 (0.6–2.4)	0.712	0.5 (0.2–0.9)	0.021
**Deep**	7 (4.0)	76 (13.0)	15 (34.1)	12.5 (4.7–33.3)	<0.001	3.5 (1.8–6.8)	<0.001
**Metrorrhagia** b	3 (8.3)	67 (16.9)	6 (31.5)	5.07 (1.10–23.38)	0.037	2.3 (0.83–6.2)	0.110
**Rash**	8 (8.5)	119 (30.1)	1 (5.6)	0.63 (0.74–5.39)	0.675	0.13 (0.17–1.03)	0.054
**Cough**	60 (34.1)	49 (22.5)	14 (31.8)	0.90 (0.44–1.82)	0.775	1.60 (0.79–3.27)	0.189
**Diarrhea** **	52 (29.5)	118 (29.4)	16 (36.3)	1.36 (0.68–2.72)	0.382	1.37 (0.71–2.62)	0.342
**Vomiting**	139 (78.9)	198 (49.9)	31 (70.4)	0.63 (0.30–1.33)	0.230	2.39 (1.21–4.71)	0.011
**Headache**	162 (92)	332 (84.1)	41 (93.2)	1.18 (0.32–4.30)	0.801	2.59 (0.77–8.63)	0.120
**Abdominal pain**	118 (67)	203 (51.2)	31 (70.4)	1.17 (0.57–2.40)	0.665	2.26 (1.15–4.46)	0.018
**Dyspnea**	67 (38.3)	59 (27.1)	24 (54.6)	1.9 (0.99–3.8)	0.053	3.23 (1.66–6.28)	<0.001
**Arthralgia**	142 (81.6)	341 (86.3)	37 (84.1)	1.19 (0.48–2.91)	0.701	0.83 (0.35–1.97)	0.684
**Jaundice**	91 (51.7)	3 (1.4)	29 (65.9)	1.80 (0.90–3.60)	0.093	138.5 (37.8–507.7)	<0.001
**Hepatomegaly**	77 (44)	7 (3.9)	26 (59.1)	1.83 (0.93–3.59)	0.075	35.28 (13.4–92.6)	<0.001
**Days of fever** c	7.4 (8.1)	4.2 (2.8)	7.59 (6.4)	0.98 (0.95–1.02)	0.540	1.32 (1.19–1.47)	<0.001
**Severe malaria** d	45 (25.6)	-	7 (15.9)	0.6 (0.2–1.3)	0.182	-	0.139
**Severe dengue** e	-	211 (36.1)	32 (72.7)	-	-	4.71 (2.37–9.34)	<0.001

a p value from Logistic regression;

b Total of 77 women in group A, 210 women in group B and 22 women in group C;

c Mean (standard deviation – SD);

d WHO malaria- Severity Criteria for Malaria from World Health Organization, 2010;

e WHO dengue- Severity Criteria for Dengue from World Health Organization, 2009;

* Bleeding was considered based on patients' history or physical examination. Superficial bleeding was defined as skin and/or mucosae bleeding and deep bleeding was defined as gastrointestinal or urinary tract bleeding. Some patients presented both superficial and deep bleeding;

** Diarrhea was defined as more than three liquid evacuations in 24 hours.

**Table 3:** Laboratory findings of hospitalized patients with *P. vivax* malaria, dengue fever and *P. vivax* malaria and dengue fever co-infection admitted to a tertiary health center (Manaus, Brazil).

Variables	*P.vivax* (A)	Dengue fever (B)	Co-infection (C)	A×C	B×C
				*p* a	*p* a
	N = 176	N = 584	N = 44		
**Parasitemia** b	2.843 (1974–4094)	-	4363 (2133–8924)	0.155	-
**Hematocrit**	30.8 (8.8)	38.0 (14.2)	31.01 (8.5)	0.473	0.002
**Leukocytes**	7.801 (5.9)	5.700 (4.2)	7.197 (4.7)	0.457	0.810
**Platelets**	115,114 (136,920)	41,824 (37,865)	69.772 (71,486)	0.055	<0.001
**Albumin**	3.5 (0.6)	3.0 (1.6)	3.38 (0.6)	0.677	0.154
**Creatinine**	1.21 (1.4)	1.0 (0.3)	1.02 (0.4)	0.214	0.951
**AST**	73.1 (98.3)	189 (543.0)	90.9 (173.6)	0.263	0.007
**ALT**	73.6 (83.5)	134 (186.0)	99.7 (192.9)	0.328	0.251
**Total bilirubin**	3.7 (5.7)	0.7 (1.0)	8.3 (13.0)	0.008	<0.001
**Direct**	1.9 (3.8)	0.4 (0.7)	3.5 (3.4)	0.033	<0.001

Mean (standard deviation- SD);

a p value from Student's T test (no variable presented non-normal distribution as assessed by the Kolmogorov-Smirnov test).

b Geometric mean; parasites/mm^3^.

AST: aspartate aminotransferase; ALT: alanine aminotransferase. Reference values: Hematocrit: 40.0–52.0%; Leukocytes: 4.0–10.8/mm^3^; Platelets: 130,000–400,000/mm^3^; Albumin: 3.5–5.0 g/dL; Creatinine: 0.7–1.5 mg/dL; AST: 0–38 IU/L; ALT: 0–44 IU/L; Total bilirubin: 0–1.3 mg/dL.

Compared to *P. vivax* mono-infected patients, the increased odds of deep bleeding in co-infected patients (OR 12.5, 95% CI: 4.7–33.3) was statistically significant (p<0.001), although platelet count was not different (Tables 2 and 3). When compared with dengue mono-infected patients, co-infected patients had a higher chance of presenting deep bleeding (OR 3.5, 95% CI: 1.8–6.8). Conversely, superficial bleeding was more frequent among dengue mono-infected patients. The overall bleeding, however, was more frequent on co-infected patients, despite significant reduction in platelet counts (Tables 2 and 3).

Regarding hepatic injury, co-infected patients had a higher chance of having hepatomegaly and clinical jaundice compared to those with malaria mono-infection, although this was not statistically significant (Table 2), despite significant increase in bilirubin levels (Table 3). When compared to dengue mono-infected patients, co-infected patients had a higher chance of presenting hepatomegaly (OR 35.28, 95% CI: 13.4–92.6) and jaundice (OR 138.5, 95% CI: 37.8–507.7), which was paralleled by significantly increased in bilirubin and AST levels (Tables 2 and 3).

Co-infected patients also had prolonged fever when compared to dengue mono-infected patients. Finally, other dengue warning signs [2], such as abdominal pain and vomiting, as well as dyspnea, were significantly more frequent among co-infected patients. Noteworthy, all four co-infected pregnant women had severe disease.

The predominant dengue serotypes in the co-infected group were DENV 2 and DENV 4, both with nine patients (33.3%). These serotypes were the most common among the dengue mono-infection group, 127 (49.6%) and 80 (31.2%), respectively.

No patient required hospitalization in the intensive care unit, and fatality rate was zero in our casuistic.

## Discussion

In an endemic area of dengue fever and vivax malaria, we found a high prevalence of the co-infection, mainly among those with malaria. In Brazil, a prospective study performed in 2009 on 132 patients with vivax malaria found 11 co-infected and the prevalence was 8.3% [10]. During a dengue outbreak in India, the prevalence of co-infection was 5.8% among all cases of fever (77 of 546) [12]. In the French Guiana, the prevalence of co-infection was 7.1% (17 of 238) among patients with dengue [11], which is similar to our results. In Pakistan, however, the prevalence found was as high as 23.2% [21]. Thus, the prevalence of co-infection may fluctuate, depending on local endemicity. In these studies, the prevalence was estimated on hospitalized patients, therefore it could not be extrapolated to the community-based level.

In our study, being co-infected resulted in a much higher chance of presenting deep bleeding as compared to both groups of mono-infected patients, suggesting a possible synergistic pathogenic mechanism, which could be related to both capillary fragility and coagulation disorders, but not the low platelet count. Bleeding is reported as an infrequent finding in malaria, despite common platelet depletion [22], [23]. Conversely, bleeding is the most feared complication of dengue fever, where in addition to platelet depletion, virus-induced endothelial and liver injury concur to the risk of coagulopathy [24], [25], [26]. In our casuistic, although bleeding was more frequent among co-infected patients, it was also frequent among mono-infected patients in both groups.

Hepatic injury was also a concern in the co-infected group, which, together with bleeding, resulted in a higher chance of dengue severity according to WHO criteria. Jaundice in malaria is mostly a result of cholestasis or intravascular hemolysis [27], while in dengue fever it has been associated with fulminant liver failure [28], [29]. Interestingly, like bleeding, jaundice is no longer considered to be a malaria severity criteria according to WHO [13]. A prospective study performed during a dengue outbreak in India, reported more frequent bleeding on co-infected patients, as well as thrombocytopenia and hepatic injury [12]. On the other hand, in the French Guiana, although co-infected patients presented more hematologic complications and hepatic injury, bleeding was uncommon [11].

A warning sign commonly used to describe severe dengue is hemoconcentration (increase in the basal hematocrit ≥20%) [30]. However, even with more severe dengue cases, our co-infected patients presented a low mean of hematocrit. An explanation for this fact can be attributed to malaria-induced anemia, a common complication in vivax malaria [31]. For this reason, the malaria clinical manifestation may be a confounder for health care professionals during the interpretation and application of dengue severity criteria, in areas where both diseases occur. The proper clinical management of co-infected patients may be compromised due to diagnostic delays or misinterpretation, and inappropriate treatment may result in fatal complications [32], [33].

Dengue warning signs, such as vomiting, abdominal pain and hepatomegaly, were very frequent in the co-infection cases. The cautious detection of these signs is of extreme importance as they characterize potential dengue severity [2]. Our findings were similar to the results reported by the study performed in the French Guiana [11], although they did not use the dengue severity criteria from WHO [2]; in both cases, the co-infected patients presented a higher frequency of warning signs and the sample had more severe cases.

In addition to classical warning signs and symptoms, dyspnea was also frequent in all groups, particularly in co-infected patients. Dyspnea is an early clinical feature of plasma leakage and, in dengue, may be the evidence of fluid accumulation of in the pleural cavity [2], [34]. In malaria, dyspnea may be an evidence of acute lung edema [35], which is one of the severity criteria for falciparum malaria [13]. In a study conducted in Timor East, one patient co-infected with falciparum malaria and dengue presented respiratory distress with radiographic findings compatible with the presentation of acute lung edema [33]. The clinical management of these cases may be difficult, as the inadequate fluid therapy for dengue treatment may induce fluid overload and large fluid effusion to the lungs.

The pregnant women had a more complicated presentation, although we could not follow up them until the end of their pregnancy. In a case series of co-infected patients from the Amazon region, pregnant women (2 of 11) presented severe acute lung edema and anemia [10]. Dengue is known to cause obstetric complications and to increase the risk of dengue severity among pregnant women [36]. In malaria, on the other hand, this association is not clear, because reported studies on the impact of *P. vivax* on pregnancy are scarce [37].

Co-infected patients presented similar days of fever as compared to malaria patients. That means that a patient with the diagnosis of dengue presenting with prolonged evolution should raise the suspicion of malaria co-infection. Our findings corroborate the results of a long case series in Pakistan, which presented longer disease duration on patients co-infected with vivax malaria and dengue [21].

No specific dengue serotype was associated to the co-infected patients, however the number of cases was not big enough to test that hypothesis.

Our study has some limitations. It was not possible to confirm dengue infection by PCR in all patients due to the time of the disease presentation and possible non-viremic periods. On the other hand, a positive IgM in patients with malaria could also reflect recent dengue infection or recent yellow fever vaccination. In addition, our results are not extendable to other healthcare settings or to community basis, since we only included hospitalized patients.

On the other hand, this study has also some strengths. This is one of few studies addressing malaria and dengue co-infection, with a considerable amount of cases diagnosed by molecular tests. Besides, this work has been conducted by the same health care team, who applied consistent selection and severity criteria throughout the duration of the study. Furthermore, the majority of the existing works are case series reports and retrospective studies, which may produce low evidence level.

### Conclusion

Malaria and dengue co-infection is a relatively common event. Being malaria the disease with easier and faster diagnosis, in areas with known endemicity, it is recommended the systematic testing for *Plasmodium* sp. on cases with acute febrile syndrome. At last, the patients with parasitological malaria diagnosis which present spontaneous bleeding must be systematically investigated for dengue, and likewise, in suspected and confirmed dengue patients presenting jaundice, *Plasmodium* sp. investigation must be performed. Besides, whenever co-infection is confirmed, we recommend a carefully monitoring for bleeding and hepatic complications, which may result in a higher chance of severity, regardless of WHO criteria.

## Supporting Information

Checklist S1 STROBE checklist.
(DOCX)
